# Biobased Food Packaging Systems Functionalized with Essential Oil via Pickering Emulsion: Advantages, Challenges, and Current Applications

**DOI:** 10.1021/acsomega.4c09320

**Published:** 2025-01-28

**Authors:** Patrícia Marques De Farias, Roberta Vieira De Sousa, Bianca Chieregato Maniglia, Melvin Pascall, Julia Matthes, Anna Sadzik, Markus Schmid, Ana Elizabeth Cavalcante Fai

**Affiliations:** † Sustainable Packaging Institute SPI, Faculty of Life Sciences, Albstadt-Sigmaringen University, Anton-Guenther-Straße 51, 72488 Sigmaringen, Germany; ‡ Food and Nutrition Graduate Program, Federal University of the State of Rio de Janeiro - UNIRIO, Av. Pasteur, 296, Urca, Rio de Janeiro, Rio de Janeiro 22290-240, Brazil; § São Carlos Institute of Chemistry, University of São Paulo - USP, Av. Trabalhador São-Carlense, São Carlos, São Paulo 00000, Brazil; ∥ Food Science and Technology, The Ohio State University, 2015 Fyffe Road, Columbus, Ohio 43210, United States; ⊥ Laboratory of Multidisciplinary Practices for Sustainability (LAMPS), Institute of Nutrition, State University of Rio de Janeiro - UERJ, R. São Francisco Xavier, 524, Maracanã, Rio de Janeiro, Rio de Janeiro 20550-013, Brazil

## Abstract

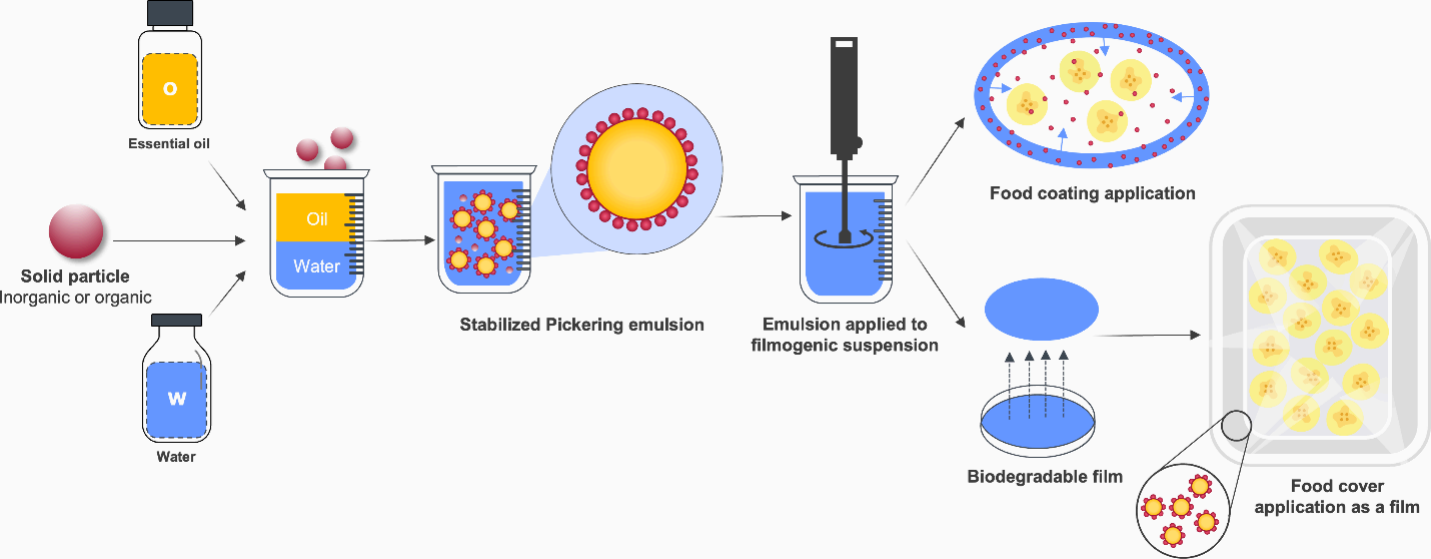

The development of innovative active food packaging is a promising strategy to mitigate food loss and waste while enhancing food safety, extending shelf life, and maintaining overall quality. In this review, Pickering emulsions with essential oils are critically evaluated as active additives for sustainable food packaging films, focusing on their antimicrobial and antioxidant properties, stabilization mechanisms, and physicochemical performances. A bibliometric approach was used to contextualize the current research landscape and new trends. Data were collected from the Web of Science and Scopus databases to find studies published between 2020 and 2024. The analysis of 51 articles shows that cinnamon, clove, and oregano are the most used essential oils, while cellulose and chitosan are the predominant polymer matrices. Pickering emulsions as stabilizers in food science represent a step forward in sustainable emulsion technology. The incorporation of essential oils into biobased films via Pickering emulsions can improve the mechanical and barrier properties, antimicrobial and antioxidant activities, and shelf life of foods. This approach offers a natural, environmentally friendly alternative to conventional materials and is in line with the 2030 Agenda goals for sustainability and responsible consumption. Recent advances show that composite particles combining polysaccharides and proteins have higher stability and functionality compared to single particles due to their optimized interactions at the interfaces. Future research should focus on developing scalable, cost-effective production methods and conducting comprehensive environmental testing and regulatory compliance, particularly for nanotechnology-based packaging. These efforts will be crucial to drive the development of safe and effective biobased active food packaging.

## 1 Introduction

The Food and Agriculture Organization (FAO) estimates that approximately one-third of the edible food produced globally for human consumption—equivalent to 1.3 billion tonnes annually—is lost or wasted. This represents not only a moral and economic failure but also an inefficient use of natural resources that must be addressed to resolve cross-cutting issues essential for achieving the 2030 Agenda.^1^

The development of active packaging, a technology designed to interact intentionally and positively with food, has emerged as a promising strategy to mitigate food loss and waste, extend shelf life, and improve food safety and quality. Food packaging systems with bioactive components can provide a variety of functions, such as moisture and oxygen absorption, antioxidant effects, free radical scavenging to mitigate oxidation, and inhibition of microbial growth. These effects can either be inherent to the base material of the packaging or introduced by the addition of bioactive compounds, such as plant extracts and essential oils that functionalize the packaging. By improving the performance of the material, this approach not only extends shelf life and reduces food loss and waste but also promotes sustainability.^2−4^

Active functionalities can be achieved through various application methods, such as using sachets with active ingredients in the packaging, incorporating active compounds into the packaging material, or coating food directly with active suspensions. The incorporation of active components into food packaging systems creates a dynamic, continuous interaction between the packaging, the food, and the external environment. The packaging serves not only as a protective barrier but also as an active system capable of releasing or absorbing certain substances such as moisture, gases, or antioxidants to create suitable internal conditions. At the same time, the food interacts with the packaging through processes such as gas exchange, moisture transfer, or the migration of aromas and compounds that can affect its freshness, safety, and quality. External environmental influences such as temperature, humidity, and oxygen content also influence these interactions and affect both the performance of the packaging and the stability of the food. This synergistic three-way interaction extends the shelf life of the product, inhibits microbial growth, and preserves its sensory and nutritional properties throughout the supply chain—from storage and transportation to marketing and final consumption.^4,5^

Food packaging is crucial for preserving safety and quality, and beyond that it has the potential to advance into active packaging, offering enhanced functionality. However, conventional petroleum-based synthetic plastics, widely used for food packaging, contribute to resource depletion, environmental pollution, and health problems due to their complex and costly recycling process, low biodegradability, and the diffusion of toxic additives and micro- and nanoplastics.^6^ To address this challenge, researchers are exploring biodegradable and biobased materials as viable alternatives for packaging solutions that not only align with sustainability goals but also meet growing consumer demand for safe, fresh food and clean labels, as well as functional packaging with minimal environmental impact.^7,8^

In the development of a new generation of food packaging, the focus is on research into environmentally friendly materials from renewable sources. Flexible biobased films, primarily composed of biopolymers such as polysaccharides, proteins, and lipids, provide a sustainable matrix for the incorporation of natural active agents.^4,9,10^ Adding essential oil (EO), bioactive compounds, or other functional ingredients to the film-forming suspension (FFS) is a viable way to enhance the antioxidant and antimicrobial properties of biobased films.^3,8^ This approach contributes to achieving the desired active properties in food packaging materials.^2^ However, EOs are highly volatile and susceptible to oxidation, light, and thermal degradation.^12,13^ In addition, when free EO is mixed directly with a FFS or added in the form of conventional emulsions, it tends to be rapidly released, which can reduce the antimicrobial or antioxidant efficacy of the packaging over the shelf life of the food product, posing a challenge for its incorporation into the film-forming process.^14,15^ To overcome this challenge, recent approaches involve encapsulating these natural compounds with colloid particles before adding them to the FFS.^16^ Therefore, the Pickering emulsion (PE), an emulsion stabilized with biopolymer particles, is the most suitable technique. PE is characterized by higher coalescence stability, stronger protection of the encapsulated compound, lower sustained release rate, safety, biodegradability, and biocompatibility.^17,18^

This review aims to provide a comprehensive analysis of Pickering emulsions with essential oils (EOPE) as innovative active additives for sustainable packaging films, highlighting their potential to improve food safety and quality. By integration of a bibliometric assessment, the current research landscape and trends in the field of EOPE-functionalized packaging are contextualized. In this study, the role of essential oils as natural antimicrobial and antioxidant agents, the stabilization mechanisms of Pickering emulsions with different particle stabilizers, and the physicochemical properties and bioactive performance of EOPE-based packaging systems are discussed. The paper briefly outlines some legal and regulatory considerations for innovative packaging solutions, emphasizing compliance with safety regulations. It also provides valuable insights into current research, highlighting the potential of these solutions as sustainable alternatives for active food packaging systems.

## 2 Methods

### 2.1 Search Strategy and Selection Criteria

In order to analyze recently published studies on EOPE, stabilizer particles, and their performance in sustainable functionalized food packaging films, bibliometric analysis was carried out. The data for this research were collected on March 23, 2024, from the Web of Science (WoS) and Scopus databases.^19,20^ The searches were conducted using the following keywords: (“Bio-based film” OR “Biobased film” OR “Active film” OR “Biopolymer” OR “Sustainable packaging” OR “Biodegradable film” OR “Active packaging film” OR “Edible film” OR “Bioactive film”) AND (“Essential oil” OR “Essential-oil”) AND (“Pickering Emulsion”). Initially, articles published in the last 5 years, from 2020 to 2024 up to the research date, were selected. The articles were then screened by title and abstract, followed by full-text analysis, to identify which studies should be included in the bibliometric analysis. To this end, exclusion criteria comprised undergraduate works, dissertations, theses, and works presented in congresses or symposia, as well as those included in Annals. In addition, studies in languages other than English, reviews, and case studies such as economic evaluations, studies that did not use essential oils, studies that did not use EO to develop PE, or studies that developed only coatings instead of films were also excluded. Thus, the articles selected for this bibliometric study included (a) original research articles and (b) research that focused on the addition of PE based on EO in the formulation of sustainable films.

## 3 Results and Discussion

### 3.1 Pickering Emulsions Containing Essential Oils as Active Additives for Sustainable Packaging Film: Contextualization and Bibliometric Assessment

Incorporating Pickering emulsions containing essential oils (EOPE) with core–shell structures can be a feasible alternative to produce active food packaging films.^21^ These films can be used to release bioactive compounds in a controlled manner, enhancing antimicrobial and antioxidant properties to fulfill the requirements of food packaging systems for storage, transportation, and preservation.^22^

**Figure 1:**
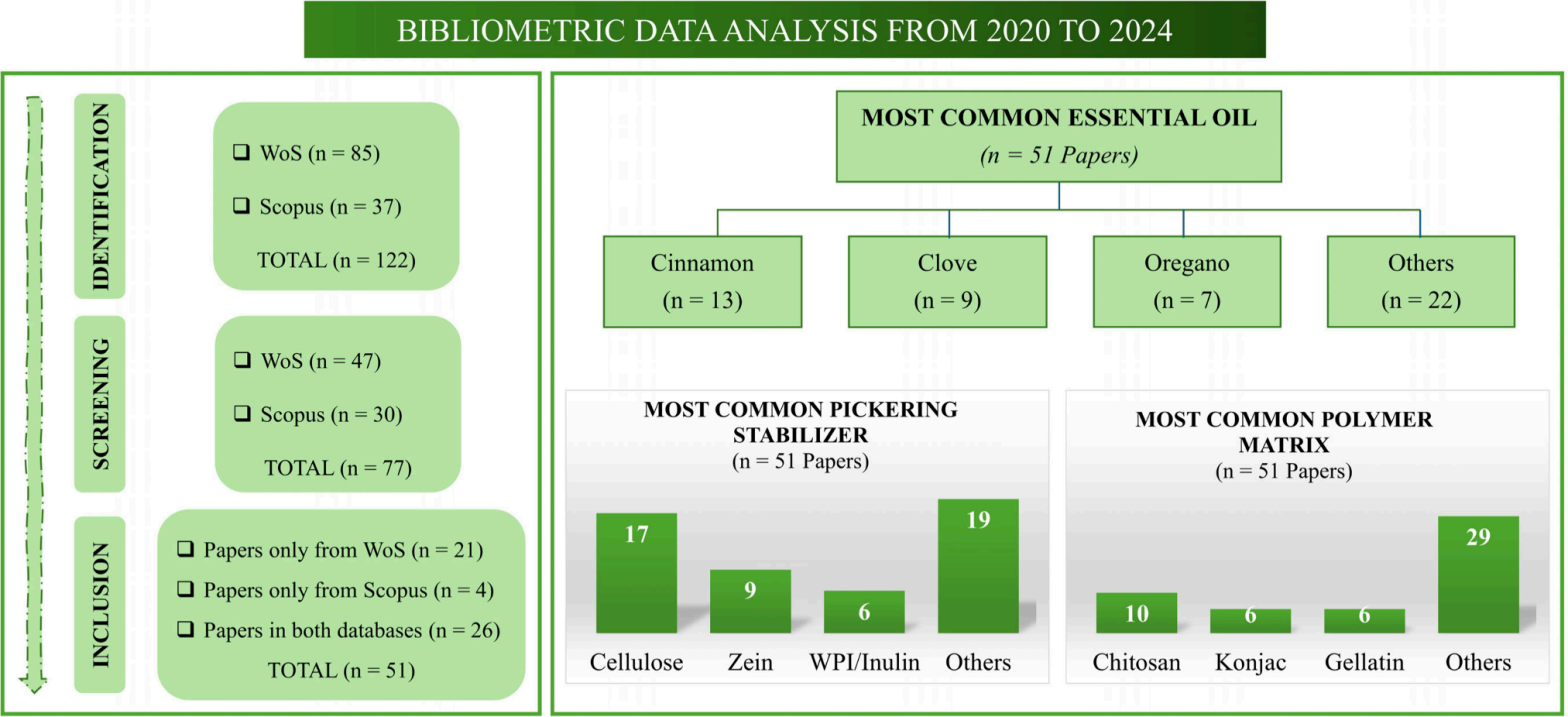
Schematic overview of bibliometric data on the addition of Pickering emulsions based on essential oils in the formulation of sustainable films.

The Web of Science (WoS) database identified 85 articles from which 47 articles were selected. Similarly, the Scopus database identified 37 articles, of which 30 were selected. After the selection criteria were applied, 51 articles, excluding duplicates, from 122 records in WoS and Scopus databases were included in the bibliometric analysis. Among the 51 articles published over the last five years and included in the bibliometric data combination, cinnamon (13) was the most studied EO, followed by clove (9) and oregano (7). In addition, the most used solid particle Pickering stabilizer was cellulose (17), followed by zein (6) and the whey protein isolate (WPI)-inulin complex (6). On the other hand, chitosan (10), konjac (6), and gelatin (6) were the most used polymer matrices to which EOPE was added (Figure 1).

### 3.2 Essential Oils and Their Potential Use for Food Safety and Quality Assurance

EOs are concentrated, volatile, hydrophobic liquids derived from various parts of aromatic plants or fruits as secondary metabolites.^23,24^ These parts include roots, stems, seeds, bark, leaves, and flowers.^23,25^ Chemically, EOs are a complex mixture of secondary metabolites such as monoterpenes, terpenes, terpenoids, aliphatic compounds, alkaloids, isoflavones, flavonoids, phenolic acids, aldehydes, and carotenoids^24,26^ with potent antimicrobial properties against foodborne pathogens such as *Salmonella* sp, *Listeria* sp., and *Escherichia coli* (*E. coli*).^27−29^ A possible mechanism of action is that these metabolites target protein groups in the bacterial membrane, altering its permeability and causing bacterial death.^30^

The United States Food and Drug Administration has classified most natural EOs and their extracts as Generally Recognized as Safe or GRAS due to their nontoxic characteristics and safety.^31^ Therefore, due to their strong, wide-spectrum activity against microorganisms, EOs are widely used in the food industry as natural preservatives to increase the shelf life of food products such as meat, fruits, vegetables, and dairy.^32^ Examples of EOs with antimicrobial and antioxidant activities are listed in Table 1.

**Table 1:** Examples of Major Antimicrobial and Antioxidant Activity of Selected Essential Oils

Essential Oil	Plant	Part of the plant used for extraction	Antimicrobial Activity (AA)	Antioxidant Activity^a^	Reference
Anise	*Pimpinella anisum*	Seeds	AA against *Trichoderma viride*, *Penicillium citrinum*, *Aspergillus niger*, *Candida albicans*, and *Staphylococcus aureus* (*S. aureus*)	Antioxidant activity of 20.42% against DPPH assay; 16.49% of radical-scavenging capacity (ABTS) method and total phenolic content of 386.37 μg of GAE/Ml	(29, 33)
Basil	*Ocimum basilicum*	Leaves	AA against *Brochothrix thermosphacta*, *Carnobacterium maltaromaticum*, *Enterococcus faecalis*, *Staphylococcus xylosus*, *Staphylococcus saprophyticus*, *Listeria innocua*, *Streptococcus salivarius*, *Serratia proteamaculans*, and *E. coli*	Effective antioxidant activity with an IC_50_ of 23.44 ± 0.9 μg/mL compared with Trolox (IC_50_ of 2.7 ± 0.5 μg/mL)	(28, 34)
Black pepper	*Piper nigrum*	Fruits	AA against *Aspergillus flavus*	Antioxidant activity assessed by β-carotene bleach assay showed lipid peroxidation of 73.6%, higher than that of ascorbic acid (15.18%)	(35)
Cinnamon	*Cinnamomum verum*	Leaves and bark	AA against *Trichoderma viride*, *Penicillium citrinum*, *Aspergillus niger*, *S. aureus*, *Listeria monocytogenes*, *E. coli*, *Salmonella typhimurium*, and *Salmonella* spp.	Antioxidant activity assessed by β-carotene bleach assay showed lipid peroxidation of 82.3% when compared to synthetic antioxidants, 2,6-di*tert*-butyl-4-methylphenol (BHT) and beta hydroxy acid (BHA) with 74.4 and 81.2%, respectively	(27, 36)
Citronella	*Cymbopogon nardus*	Leaves	AA against *Candida albicans*, *S. aureus*, *Staphylococcus epidermidis*, *Streptococcus mutans*, and *Salmonella typhimurium*	Mild antioxidant activity with an IC_50_ of 274 ± 2 mg/g compared to BHT and ascorbic acid (IC_50_ of 8029 ± 1 mg/g and 5137 ± 4 mg/g, respectively)	(37, 38)
Clove	*Eugenia caryophyllata*	Flowers	AA against *S. aureus* and *E. coli*	DPPH scavenging activity IC_50_ ranged from 15.80 to 108.85 μg/mL, depending on flowering stages	(39, 40)
Ginger	*Zingiber officinale*	Root	AA against *S. aureus* and *E. coli*	Antioxidant activity assessed by β-carotene bleach assay showed lipid peroxidation of 66,5%, lower antioxidant activity than that shown by BHT and BHA (74.4 and 81.2%, respectively)	(36,41)
Grapefruit	*Citrus paradisi*	Peel	AA against *E. coli* and *Leuconostoc mesenteroides*	Antioxidant activity assessed by ABTS and DPPH showed 24 mg Trolox Equivalent (TR)/ml CEO and 15 mg TR/ml CEO, respectively	(42)
Lavender	*Lavandula angustifolia*	Flowers	AA against *Candida tropicalis* and *Pseudomonas aeruginosa*	Moderate antioxidant activity of 29.08 ± 0.99% of DPPH scavenging capacity	(43)
Lemon	*Citrus limon*	Peel	AA against *E. coli* and *Leuconostoc mesenteroides*	Antioxidant activity assessed by ABTS and DPPH showed 7 mg TR/ml CEO and 23 mg TR/ml CEO, respectively	(42)
Lemongrass	*Cymbopogon citratus*	Leaves	AA against *Candida krusei*	Antioxidant activity of 853.0 ± 1.13 μg TR/ml LEO (i.e., 84.0 ± 0.1%) of DPPH scavenging capacity	(44)
Mandarin	*Citrus reticulata*	Peel	AA against *S. aureus*	Antioxidant activity assessed by ABTS and DPPH showed 31 mg TR/ml EO and 17 mg TR/ml CEO, respectively	(42, 45)
Marjoram	*Majorana hortensis* Moench	Leaves	AA against *P. aeruginosa*	Mild antioxidant activity with an EC_50_ value of 54.01 mg/mL EO of DPPH scavenging capacity	(46, 47)
Oregano	*Origanum vulgare* L.	Leaves	AA against *Campylobacter* spp, *Shewanella putrefaciens*, and *Vibrio vulnificus*	Effective antioxidant activity with an EC_50_ value of 7.450 mg/mL EO of DPPH scavenging capacity	(47−50)
Rosemary	*Rosmarinus officinalis*	Leaves	AA against *Listeria monocytogenes* and *S. aureus*	Moderate antioxidant activity of 28.76 ± 2.68% of DPPH scavenging capacity	(43, 51)
Thyme	*Thymus vulgaris*	Leaves	AA against *Pseudomonas fluorescence* and *E. coli*	Effective antioxidant activity with an EC_50_ value of 0.944 mg/mL EO of DPPH scavenging capacity	(46, 47)

a Abbreviations: DPPH, 2,2-diphenyl-1-picrylhydrazyl free radical-scavenging capacity; ABTS, 2,2′-azinobis-3-ethylbenzothiazoline-6-sulfonic acid; GAE, gallic acid equivalent; IC_50_ or EC_50_, concentration of extract necessary to neutralize 50% of initial concentration of free radicals.

### 3.3 Pickering Emulsions and Particle Stabilizers

Emulsions are colloidal systems composed of at least two immiscible fluids, with one dispersed in the other as small droplets.^17,18^ Traditionally, emulsion systems have been composed of oil and water, which can form three types of emulsions: oil-in-water (O/W), water-in-oil (W/O), and complex or multiple emulsions, with O/W emulsions being the most common.^52^ However, due to the high surface energy between the two immiscible phases, emulsion systems are considered thermodynamically unstable and susceptible to coalescence over time. Therefore, to ensure that the emulsion remains stable, a surface-active agent or stabilizer such as a chemical surfactant is needed.^53^ Most of the surfactants commonly used in traditional emulsions, such as hexadecyltrimethylammonium bromide, benzalkonium chloride, alkylbenzene linear sulfonate,^54^ Tween 20, Tween 80, Span 20, and Span 80,^55^ present a considerable environmental challenge, particularly in terms of soil and water pollution. Their nondegradability in the environment gives rise to considerable concerns regarding sustainability and the health of ecosystems. Additionally, the potential adverse health effects of these emulsifiers are dependent on the dosage required to stabilize the emulsion.^54,56^ As demand for more sustainable systems increases, so does interest in using natural and clean-label ingredients instead of synthetic surfactants.^57^

**Figure 2:**
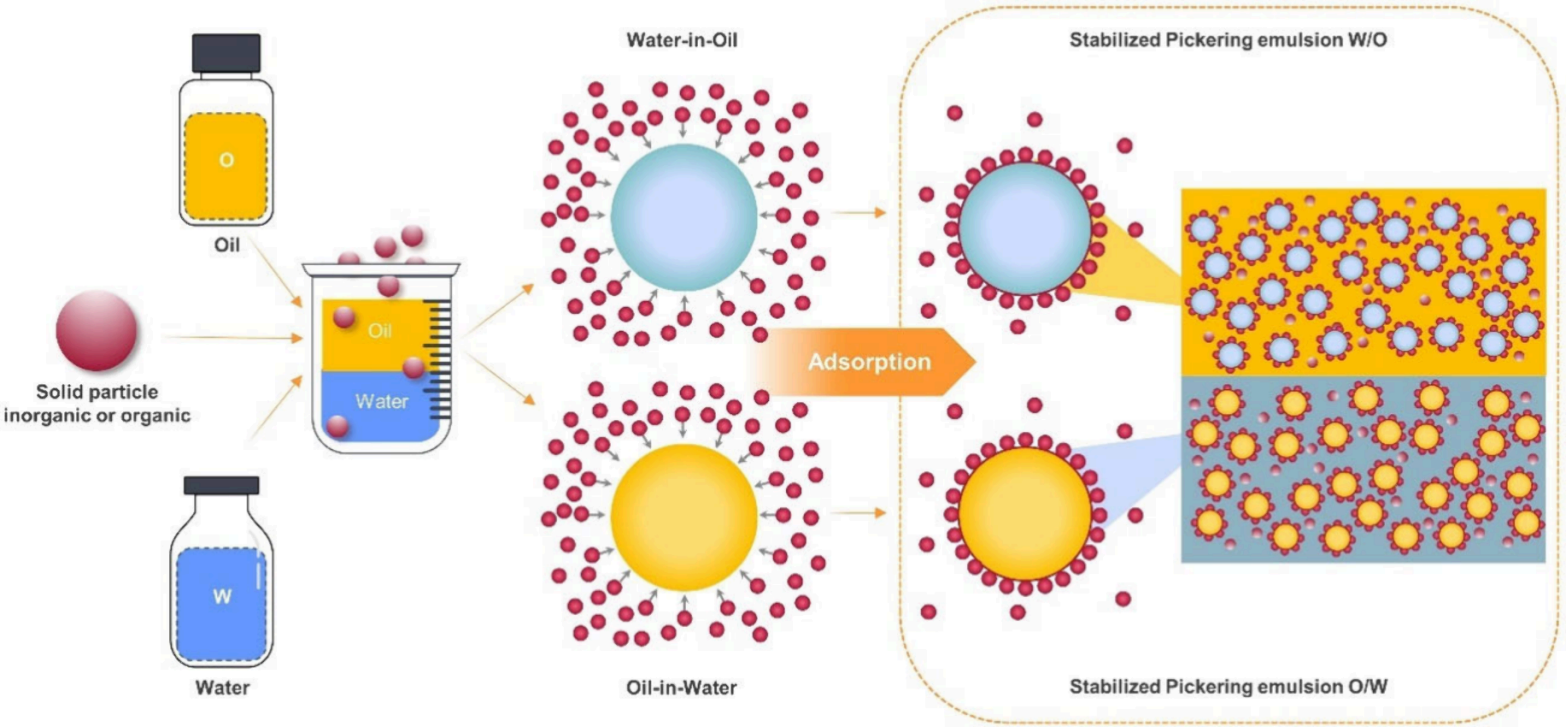
Schematic representation of particle stabilizers in Pickering emulsions.

PEs are stabilized by solid particles (nano- and microparticles) and have been demonstrated to have no toxic effects, particularly when developed using food-grade biopolymer particles,^15,16^ lower cost, and easier recovery properties compared to conventional surfactants (Figure 2). Solid colloidal particles can replace surfactants to stabilize oil/water interfaces.^53,57,58^ One significant advantage of using these particles is that they can irreversibly adsorb to an oil/water interface and prevent droplet coalescence. This property means that once a particle is at an interface, it will not detach from it spontaneously, even if there are temperature changes. This outstanding stability and encapsulation efficiency are highly desirable and make PE a suitable technology for the protection and controlled release of bioactive compounds that are fixed in a given matrix.^13,59^

Currently, there are several ongoing research studies on the manufacturing strategy of food-grade particles that can stabilize PE systems.^60^ To be feasible as a PE stabilizer, the particle must be partially wettable by both the continuous and dispersed phases of the system, remain insoluble, present a low surface charge, and be much smaller than the emulsion size.^53^ These particles are classified into single and composite particles. Although each solid particle has unique inherent characteristics, such as wettability, size, and shape, which affect the necessary processing conditions differently, single solid particles used as stabilizers for PE can be categorized into three groups based on their origin. These three groups are biological, inorganic, and organic particles. (i) Biological particles are derived from natural biopolymers and are usually renewable, biodegradable, and biocompatible. Examples include cellulose nanocrystals, carboxymethyl chitosan, pea protein nanoparticles, starch, zein, pectin, and gliadin nanoparticles. These particles are particularly attractive for the development of sustainable food packaging due to their functional properties such as film-forming capacity, mechanical reinforcement, antioxidant, and antimicrobial activity. In contrast, (ii) inorganic particles are synthesized from minerals and other nonorganic sources, such as hydrophilically modified silica, halloysite clay nanotubes, kaolinite, and shigaite-like layered double hydroxide particles. These particles offer excellent thermal stability and barrier properties, making them good candidates for improving the structural integrity and shelf life of packaging materials. (iii) Organic particles are usually synthetic polymers such as poly(caprolactone) block-poly(ethylene oxide) diblock copolymers and oligoimide particles. The choice of the appropriate particle type depends on the specific functional requirements of the packaging system as well as sustainability, regulatory compliance, and end-of-life disposal considerations.^56^

Due to their hydrophilic nature and other inherent properties, certain food-grade biopolymer particles, such as starch, chitosan, and cellulose, exhibit low surface activity at the oil/water interface, which reduces their applicability.^60,61^ To overcome such limitations of single particles, composite particles have become a popular area of research, since they generally exhibit superior functionality, particularly in terms of wettability and adsorption behavior, resulting in long-term stability and providing controlled release, making them more useful.^62,63^ Composite particle formations can be achieved using proteins, polysaccharides, phenolic compounds, or lipids through solvent mediation and interactions based on the functional properties of biopolymers, particles, and conditions. Examples of composite particles include the following: (I) water-soluble protein-polysaccharide particles, pea protein isolate-high methoxyl pectin and whey protein isolate-dextran; (II) water-insoluble protein-polysaccharide particles, zein-gum arabic and zein-propylene glycol alginate; (III) water-soluble protein-water insoluble polysaccharide particles, soy protein isolate-bacterial cellulose nanofibers, soy protein isolate-chitosan, and pea protein isolate-chitosan; (IV) water-insoluble protein-water insoluble polysaccharide particles: zein-chitosan and gliadin-chitosan.^64^

### 3.4 Properties and Applications of Pickering Emulsions Containing Essential Oils Functionalized Packaging

Among the 51 papers included in this bibliometric analysis published in the last five years, cinnamon, clove, and oregano EO were the most studied, with 13, 9, and 7 studies, respectively. The growing interest in cinnamon and clove oils is due to their remarkable bioactive properties, which make them attractive candidates for use in active food packaging. Both oils are recognized as safe food additives and are known for their strong antioxidant capacity and broad-spectrum antimicrobial activity against a variety of food-borne microorganisms. Their classification as “Generally Recognized as Safe” (GRAS) increases their appeal as a natural alternative to synthetic preservatives, responding to consumer demand for environmentally friendly packaging solutions.^4,66,68,69^ The incorporation of essential oils such as cinnamon, clove, and oregano into packaging films has the potential to improve both bioactivity and functional properties. These oils can contribute to improved mechanical strength and reduced water vapor permeability, which could help protect food by limiting moisture transfer and microbial contamination.^4,65,69,70^

The incorporation of PE based on cinnamon EO (CEO) into collagen films, coupled with oxidized mulberry extract (OME) as a functional enhancer, significantly improved the mechanical and barrier properties of the films. The tensile strength increased from 79.18 to 106.35 MPa, which increased the durability of the films, while the water vapor permeability decreased from 2.82 to 2.30 × 10^–11^ g/(m s Pa) and the water absorption was reduced from 524.57% to 201.28%, reflecting better moisture resistance. The films also exhibited improved light-blocking, antioxidant, and antimicrobial properties and effectively inhibited *Escherichia coli* and *Pseudomonas fluorescens*. Applied to fish fillets, the CEO PE/OME film extended the shelf life of the product by 4 days and preserved its quality and safety. The film also served as a freshness indicator: it changed color from red on day 0 to black-green on day 12 (Δ*E* > 5), allowing a visual assessment of the freshness of the fish. These results underline the potential of CEO-based Pickering emulsion collagen films as multifunctional packaging materials that combine longer food preservation with real-time freshness monitoring.^4^

Wu et al.^65^ reported that cinnamon EOPE was incorporated into films made from chayote tuber starch, resulting in significant changes in film properties. As the concentration of cinnamon EOPE increased, the tensile strength (TS) decreased from 5.44 to 2.59 MPa, while the elongation at break (EB) increased, reaching 70.8% at 4% EOPE. This decrease in TS is due to the disruption of the structural integrity of the film, which impaired the formation of hydrogen bonds between the starch molecules. The increase in the EB is probably due to the plasticizing effect of the free cinnamon EO, which increases the flexibility of the polymer chains. The water resistance of the films improved with the addition of cinnamon EOPE, especially at concentrations below 2%. This improvement was attributed to the formation of hydrogen bonds between the hydrophilic starch matrix and the hydrophobic particles. The moisture content (MC) of the films decreased slightly from 21.20% to 19.49% due to the hydrophobic properties of cinnamon-EO. However, at higher concentrations (3% and 4%), a slight increase in water solubility was observed, which is probably due to the leaching of the EO. The water vapor permeability (WVP) of the films was reduced by the addition of cinnamon EOPE, with the lowest value (1.24 × 10^–10^ g/(m s Pa)) observed at 2%. This was attributed to the increased resistance to the movement of water molecules due to changes in the internal structure of the film. These results suggest that cinnamon EOPE can significantly improve the functional properties of starch-based films, contributing to their effectiveness in food packaging.

Yao et al.^67^ investigated the effects of cinnamon EOPE stabilized with zein and carboxymethyl tamarind gum on the properties of hydroxypropyl methylcellulose films, also examining the influence of the degree of carboxymethylation. The influence of packaging on the shelf life of cherry tomatoes was also investigated. The droplet size of cinnamon-EOPE decreased significantly from about 93.03 to 10.59 μm as the degree of substitution of carboxymethyl-tamarind gum increased, which promoted a more uniform distribution of droplets in the film matrix. The incorporation of cinnamon EOPE into the hydroxypropyl methylcellulose films resulted in a significant increase in TS from 8.46 to 25.41 MPa, while the water vapor permeability decreased from 6.18 × 10^–10^ to 4.24 × 10^–10^ g/(m s Pa), indicating improved barrier properties. The films also showed improved UV protection without compromising transparency, making them ideal for use in food packaging. The films enriched with cinnamon EOPE also showed antibacterial activity against *Escherichia coli* and *Staphylococcus aureus* compared to pure HPMC films. The antioxidant activity was also significantly increased. The EOPE-added films helped to reduce the weight loss of cherry tomatoes compared with unpackaged control tomatoes while slowing the decrease in total soluble solids and titratable acidity, indicating improved preservation. The films contributed to an extended shelf life of the tomatoes by effectively delaying spoilage over a 20 day storage period.

In a study by Zhao et al.,^68^ antimicrobial films were produced by incorporating clove EOPE into a matrix of potato starch and poly(vinyl alcohol). The clove EOPE exhibited a zeta potential of −21.7 mV, a droplet size of 186 nm, a polydispersity index of 0.104, and an encapsulation efficiency of 57.9%. These results confirm the successful formation of the emulsion. The clove EOPE was uniformly distributed in the matrix of potato starch and poly(vinyl alcohol), resulting in a smooth and homogeneous film structure. The mechanical properties of the films were significantly affected by the hydrogen bonding and electrostatic interactions between the emulsion and the matrix, resulting in a decrease in TS from 22.4 to 6.80 MPa and a decrease in EB from 375.3% to 91.6%. The films were used for the preservation of pork and were found to provide an extended preservation time of 6 to 10 days and exhibit greater inhibition of *Escherichia coli* compared to *Staphylococcus aureus*. The incorporation of clove EOPE into the films showed a significant antimicrobial effect and potential to improve the preservation of pork.

Bangar et al.^69^ explored the potential of cellulose nanocrystals (CNCs) derived from kudzu (*Pueraria montana*) vine, combined with clove essential oil Pickering emulsions (EOPE), to enhance the properties of starch-based films. The incorporation of clove EOPE into a composite film of pearl millet starch and kudzu CNCs led to significant improvements in mechanical properties, with TS increasing from 4.02 to 16.2 MPa, Young’s modulus rising from 84 to 398 MPa, and EB decreasing from 48.9% to 30.4%. Moreover, the composite film demonstrated excellent antimicrobial activity against *Staphylococcus aureus* and *Escherichia coli* and was highly effective in extending the shelf life of red grapes, maintaining freshness for up to 15 days at 5 °C.

In another study,^70^ the development of films based on konjac glucomannan activated by oregano EOPE stabilized with zein-pectin nanoparticles was investigated. The results showed that the hydrophilicity and hydrophobicity of the konjac glucomannan films can be modulated by adjusting the oregano-EOPE concentration, which has a significant effect on the mechanical and barrier properties of the films. The films with oregano-EOPE to konjac glucomannan ratios of 50:50 and 60:40 exhibited the highest tensile strength (30.98 MPa) and water contact angle (93.56°), respectively, indicating better mechanical properties and higher hydrophobicity. The films showed an effective slow release of the EO for up to 21 days, which underlines their potential for food preservation. The water vapor permeability of the pure konjac-glucomannan films was 5.30 × 10^–1^ g/(m s Pa), while the oregano-EOPE-added films, especially those containing 60% oregano-EOPE content, showed a significant decrease in WVP to 1.16 × 10^–1^ g/(m s Pa), indicating an improved water vapor barrier. Increasing the oregano EOPE concentration from 0% to 60% resulted in a steady decrease in WVP, which was attributed to the formation of a more compact network and a higher crystallinity of the films. The addition of oregano EOPE to konjac-glucomannan films resulted in suitable films for fruit preservation.

Some recently published studies on the properties of EOPE-functionalized films applied to various foods are listed in Table 2.

**Table 2:** Influence of EOPE Functionalization on Sustainable Food Packaging’s Mechanical, Physical, Thermal, Antimicrobial, and Antioxidant Properties^a^

Essential Oil	Polymer Matrix	Pickering Stabilizer	Antimicrobial effect	Antioxidant Activity	Food Application	Reference
Animal-Source Foods						
*Alpinia galanga*	PVA/acetylated pullulan	Soybean protein isolate-chitosan	EOPE contributed to the antibacterial activity and showed a dose-dependent effect; moreover, the antibacterial effect on *S. aureus* was more effective than that on *E. coli*	The inclusion of EOPE demonstrated a strong DPPH and ABTS scavenging ability, and the strength of the antioxidant ability showed a dose dependence with the addition of the emulsion	Extend chicken’s shelf life and maintain sensory parameters such as pH and color	(71)
Cinnamon	Chayote tuber starch (CTS)	Zein-pectin nanoparticle	Composite films incorporated with at least 2% of EOPE showed stronger antibacterial activity against *S. aureus* and *E. coli*	The DPPH radical scavenging of the films increased significantly from 9.51% to 56.92% as the EOPE content increased from 0% to 4%, respectively	The addition of 2% EOPE significantly slows the formation of TVB-N, delaying the degradation of ground beef and extending its shelf life	(65)
	Chitosan	Cellulose nanocrystal	The films containing EOPE exhibited antimicrobial activity against *E. coli* and *S. aureus*. The inhibition zones of the films increased as the EO content was increased	NA	The preservation of pork pieces was enhanced while maintaining their structural stability	(72)
	Collagen	Soy protein isolate-chitosan	Inhibiting the growth and reproduction of *E. coli* and *Pseudomonas fluorescens*	DPPH radical scavenging of 50.17% was achieved with the addition of the EOPE	Fish preservation and freshness indicator through pH sensitivity	(4)
Cinnamon and perilla	Chitosan nanoparticles/Anthocyanidin/Collagen	Collagen	At the end of storage, films containing EOPE exhibited the lowest total bacterial count (5.89 ± 0.01 l g (CFU/g)) compared to the control group (10.85 ± 0.01 l g CFU/g)	NA	Preservation of fresh red sea bream fillets and delayed lipid oxidation and proteolysis. The shelf life of fillets was extended from 6 to 14 days	(73)
	Anthocyanidin/Chitosan	Collagen	NA	The incorporation of various concentrations of EOPE resulted in greater antioxidant activity, as measured by DPPH scavenging activity, compared to the control group samples (i.e., EOPE 0%)	The freshness of packaged fish fillets was extended in terms of thiobarbituric acid (TBA) levels	(74)
Clove	Potato starch/polyvinyl alcohol (PVA)	WPI-inulin	The film showed potent inhibition zones against *S. aureus* and *E. coli*. As the EO concentration in PE increased, the diameter of the inhibition zones increased more significantly	The films added with EOPE exhibited great DPPH scavenging activity; however, the additional amount of EOPE did not noticeably alter the antioxidant activity. Therefore, no dose dependence was observed	Extending the preservation period of pork meat from 6 to 10 days	(68)
	Gelatin/agar	Copper-modified zinc oxide nanoparticles	The EOPE film exhibited antimicrobial activity against both Gram-positive (*Listeria monocytogenes*) and Gram-negative (*E. coli*) bacteria. However, it demonstrated slightly greater antibacterial activity against *L. monocytogenes* than against *E. coli*	The pure gelatin/agar-based film exhibited a modest ∼6% DPPH and ∼33% ABTS free radical scavenging; however, the incorporation of EOPE increased this free radical scavenging activity to ∼33% and ∼61% against DPPH and ABTS, respectively	The use of EOPE film to wrap pork belly resulted in delayed bacterial growth, as evidenced by the total aerobic bacterial count (TABC). Even after 8 days, the TABC value of the test group did not exceed 6.7 log CFU/g	(75)
Grapefruit	Lotus seed drill core powder starch	Corn nanostarch	Adding 20% EOPE exerted antibacterial activity against *E. coli* and *S. aureus*; the inhibition zone diameters of *E. coli* and *S. aureus* increased significantly to 11.71 ± 0.03 mm and 10.25 ± 0.33 mm, respectively	NA	Prolong the shelf life of refrigerated pork meat pieces, based on TVB-N and TBARS reduced content	(66)
Lavender	Gelatin	Gelatin	The film containing EOPE exhibited antibacterial activity against *S. aureus* and *E. coli*; additionally, the formulation demonstrated controlled release of the EO, indicating potential for long-term preservation	All films developed with EOPE exhibited a high ABTS+ scavenging capacity	Real-time monitoring and maintenance of shrimp freshness	(76)
Nutmeg	Low-density polyethylene (LDPE)	Inulin-WPI	The interaction between LPDE/PE slowed release of the active ingredients compared to the free/coarse emulsion or the nanoemulsion; the decrease in *E. coli* growth suggests an increase in bacterial inhibition due to the higher concentration of EOPE	The concentration of EOPE significantly increased the DPPH-scavenging activity of the film, with values ranging from 38 to approximately 66%	Preserve the quality and extend the shelf life of Tilapia fish fillets, based on TBARS and TBV-N values	(77)
*Thymus vulgaris*	Tapioca starch/polyvinyl alcohol (PVA)	Cellulose nanocrystals	The composite film containing 20% EOPE exhibited bacteriostatic properties for up to 8 days, as determined by the total viable counts (TVC)	NA	Composite film with 20% TEVO PE extends fish fillet freshness for up to 8 days	(78)
*Zataria multiflora*	Chitosan	Zein	The microbiological analysis was performed by total viable count (TVC); chitosan coatings with EOPE showed stable and long-term antimicrobial activity compared to the coating with free EO	NA	EOPE delayed chemical and microbial spoilage, extending the shelf life of salmon (*Salmo trutta*) stored at refrigerator temperatures	(79)
Plant-Source Foods						
Anise	Corn starch/cassia gum	Sodium starch octenyl succinate	EOPE-incorporated composite films showed stronger dose-dependent antibacterial activity against *S. aureus* and *E. coli*	The DPPH radical scavenging ability of the composite films significantly increased by 18.45% with the addition of the EOPE with an oil/water phase ratio of 6:4. A similar improvement was observed in the scavenging ability of the composite membrane against ABTS radicals	Delay the browning and spoilage and maintain the original quality of *Agaricus bisporus* (mushroom)	(80)
Basil	Corn starch	Cellulose nanofiber	NA	NA	Maintain quality and prolong the shelf life of mandarin oranges	(81)
Cinnamon	Hydroxypropyl methylcellulose (HPMC)	Zein-carboxymethyl tamarind gum	Films containing EOPE showed stronger antimicrobial activities against *E. coli* and *S. aureus*	The addition of EOPE to the film matrix increased the DPPH scavenging activity by 52.00%, indicating its antioxidant ability	Increasing the shelf life of cherry tomatoes	(67)
Clove	Pearl millet starch/kudzu (*Pueraria montana*) cellulosic nanocrystals (CNC)	Kudzu CNC	Films with EOPE exhibited remarkable antimicrobial properties against *S. aureus* and *E. coli*	NA	Composite films containing EOPE were found to be highly effective in extending the shelf life of red grapes up to 15 days at 5 °C; the films maintained the weight, firmness, and soluble solids of the grapes	(69)
Ginger	Carboxymethyl cellulose/polyvinyl alcohol (PVOH)	Oleic acid	The EOPE-incorporated films showed antimicrobial effects that increased with increasing concentration of EOPE; the 3% EOPE film showed the highest antifungal activity against *Penicillium digitatum*	The incorporation of EOPE led to a dose-dependent increase in polyphenolic compounds in the films; furthermore, the films with added EOPE (0.5–3%) exhibited significantly higher antioxidant activity in DPPH scavenging effects (10.16%, 17.16%, and 21.09%, respectively)	Increasing the shelf life of bread slices from 4 to 30 days	(82)
Lemongrass	Chitosan	Cellulose nanofibers	The addition of EOPE to the film suppressed the fungal activity of *Botrytis cinerea* in inoculated tomatoes in an in vivo antifungal test	NA	The controlled weight loss and fungal contamination during storage increased the shelf life of tomatoes	(83)
*Litsea cubeba*	Gelatin/ZnO nanoparticles	Pectin-β-cyclodextrin	The film exhibited remarkable antimicrobial properties against *Colletotrichum gloeosporioides* and *Listeria monocytogenes*	The addition of EOPE to the film matrix resulted in up to 73% DPPH radical scavenging activity	The optimized films improved the postharvest quality and reduced the anthracnose of fresh mango samples during storage	(52)
Oregano	Konjac glucomannan	Zein–pectin nanoparticle	The EOPE-functionalized film resulted in significant antibacterial effects, inhibiting the growth of *E. coli* at the initial stage more effectively than that of *S. aureus*	The film’s oxidation resistance was found to increase with a higher concentration of EOPE. In the DPPH assay system, the 75% EOPE film exhibited the highest DPPH radical scavenging activity and an antioxidant capacity comparable to that of vitamin C.	Strawberries wrapped in films showed softening instead of spoilage, confirming the films’ ability to preserve freshness	(70)

a NA: not assessed.

**Figure 3:**
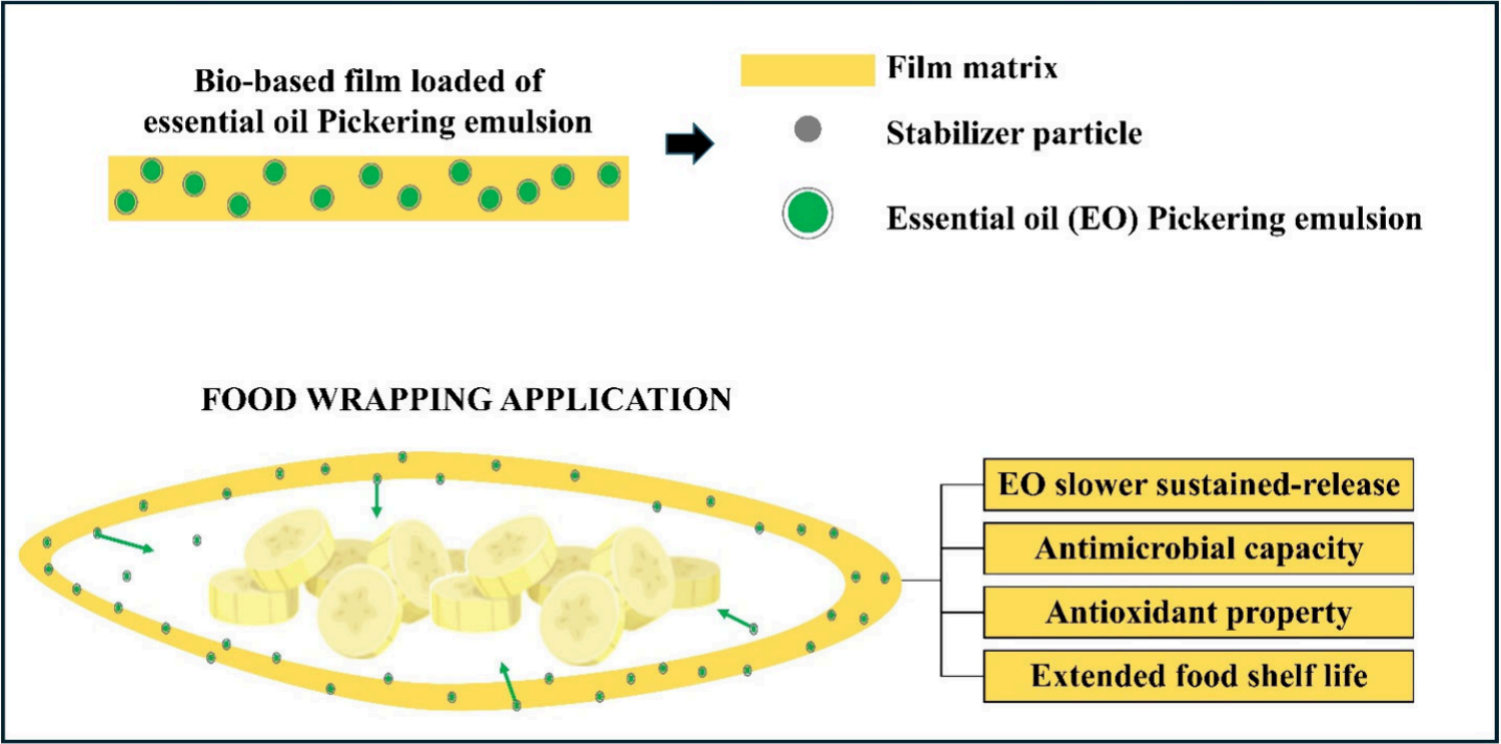
Schematic representation of the advantages of using biobased packaging for food with added essential oil by Pickering emulsion.

As Table 2 shows, EOs are interesting candidates for use as natural antioxidant and antimicrobial additives for the functionalization of food packaging.^31,84^ However, they are usually highly sensitive to oxidative conditions and elevated temperatures, which can trigger chemical instability, volatilization, oxidation, and susceptibility to degradation,^14,32,85^ thereby posing several challenges for their incorporation into the film-forming process.^70,86^ However, incorporating EO into biobased films through PE has been shown to enhance their stability while providing multiple functional benefits, including improved antioxidant capacity, antimicrobial activity, and extended shelf life of packaged products (Figure 3).

Ongoing research continues to investigate various methods for developing food-grade particles that stabilize Pickering emulsions. From the recent studies in Table 2, individual particles such as corn nanostarch,^66^ cellulose nanocrystals^72,78^ or nanofibers,^81,83^ zein^79^ and collagen^73,74^ are more commonly used as PE stabilizers, although the benefits of composite particles have been reported in the literature. Some work has shown that the combination of polysaccharides and proteins is more useful, as they generally have higher long-term stability.^60,61^

Polysaccharides generally have hydrophilic properties and, therefore, have limited surface activity at the oil–water interface. An effective strategy to solve this problem is the combination of polysaccharides and proteins, which optimizes the behavior at the interface by balancing their hydrophilic and hydrophobic properties, thus increasing the stability and functionality of emulsified systems.^60,61^ On the other hand, encapsulation by the PE technique may reduce the antibacterial activity of the EOs to a certain extent due to the controlled release of the encapsulated active ingredients. However, this controlled release may also help to maintain functional activity during long-term storage.^21,65^

Li et al.^60^ modified pea protein and chitosan to produce composite particles that effectively stabilize a Pickering emulsion with a high-volume fraction of oil phase (75% corn oil). The incorporation of polysaccharides such as chitosan into proteins such as pea protein significantly improved the emulsifying properties and resulted in a stable emulsion with a low susceptibility to coalescence and phase separation. This stability is attributed to the strong interactions and steric repulsion by the polysaccharide-protein complexes at the oil–water interface, which improves the structural integrity of the emulsion. The optimized stability of these emulsions allows them to be used as effective fat substitutes in various foods, such as pork sausages, without compromising texture or sensory properties.

In addition, the use of such emulsion systems allows the encapsulation and controlled release of bioactive compounds, which improves their bioavailability and chemical stability during processing and storage.^61^ This dual function of improving emulsion stability and providing nutrients makes Pickering emulsions of polysaccharides and proteins highly beneficial for the development of healthier foods with improved quality characteristics. The blending of polysaccharides and proteins in Pickering emulsions is therefore a promising strategy for innovative food packaging and formulation solutions.^61^

### 3.5 Legal Requirements for the Market Entry of Innovative Packaging Concepts Containing Pickering Emulsion

Due to their antioxidant and antimicrobial potential, the PE described in this paper are promising resources for the design of innovative active packaging concepts. However, before an active packaging concept can be successfully introduced to the market, it must be safe and comply with regulatory requirements.^89^ In Europe, the central regulation governing materials that come into direct contact with food is regulation (EC) No. 1935/2004. This regulation aims to oversee materials such as packaging that directly touch food and their introduction to the market while also ensuring the protection of human health (Reg (EC) No. 1935/2004). The European Union allows the use of active packaging as long as it is safe, effective, and complies with all the requirements of regulations No. 1935/2004 and No. 450/2009, which concerns “active and intelligent materials and articles intended to come into contact with food” (Reg (EC) No. 450/2009).

As described in this paper, nanoparticles can be used as stabilizers for PE. The use of substances in nanoform is not explicitly mentioned in regulation (EC) No. 1935/2004. Nevertheless, regulation (EU) No. 10/2011, which specifically regulates plastic materials in direct contact with food, allows their use as long as they are listed in and used according to a positive list provided within the regulation (Reg (EU) No. 10/2011). Nanoform substances have different chemical and physical properties compared to their conventional counterparts, mainly due to their reduced particle size, larger surface area, and higher reactivity. Recognizing these differences, Reg (EU) No. 10/2011 requires all nanoscale substances to undergo a case-by-case risk assessment by the European Food Safety Authority (EFSA). This tailored assessment ensures a comprehensive understanding of the unique toxicological profiles associated with nanomaterials before they are approved for food contact applications. The assessment process includes several crucial steps: (i) a detailed characterization of physicochemical properties, including particle size, morphology, and surface chemistry; (ii) a toxicological evaluation to assess potential mutagenic, carcinogenic, and reproductive toxicity; (iii) migration testing to quantify the transfer of nanoparticles from the packaging into the food matrix; (iv) an exposure assessment to establish safe consumption levels. Only nanoparticles that are classified as safe after this rigorous assessment will be included in the Union list of authorized substances, subject to certain conditions for their use.

## 4 Conclusion and Perspectives

Pickering emulsions, stabilized by various solid particles and macromolecules, have emerged as a promising alternative for incorporating and ensuring the sustained release of essential oils into biobased polymer matrices.

To ensure the integrity of the film during storage and transportation and to prevent the growth of microorganisms, an effective approach is to increase the microstructural density of the films by improving the polymer matrix cross-linking. This improvement can be accomplished through physical, chemical, or enzymatic methods. Chemical modifications involve the use of additives to cross-link the polymer chains. Enzymatic modifications catalyze the binding of natural mediators to the side chain groups of the polymer matrix molecules.^4^ Alternatively, low-pressure plasma (LPP) provides a nonthermal option for enhancing the adhesion of active ingredients and the physical properties of packaging materials. Treatment with LPP has been shown to improve material properties by introducing new functional groups on the surface, thereby enhancing mechanical and hydrophilic properties.^77^ Therefore, biobased food packaging systems functionalized with EOPE show significant potential for improving food preservation. These systems enhance antimicrobial activity against common food contaminants, including *Listeria monocytogenes*, *S. aureus*, and *E. coli*. They also exhibit antioxidant properties, including ABTS and DPPH radical scavenging abilities. Additionally, they promote sustainability by reducing the reliance on synthetic materials and effectively extending the shelf life of packaged foods.

The successful implementation of EOPE-based packaging in commercial applications requires a multidisciplinary approach that addresses critical challenges such as scalability, regulatory compliance, and environmental impact. To bring EOPE film production from the laboratory to industry, formulation and processing techniques must be optimized to ensure cost efficiency and compatibility with existing packaging machinery. In addition, the development of standardized protocols to assess the migration of active ingredients and nanoparticles in food matrices is essential to ensuring consumer safety and regulatory approval. Collaboration with regulatory authorities and industry representatives can facilitate the creation of safety guidelines and speed up the commercialization process.

It is anticipated that the commercial use of antimicrobial and antioxidant materials derived from natural resources will increase, leading to enhanced safety and longer shelf life. Thus, future research endeavors should prioritize the incorporation of intelligent systems with active compounds through the utilization of nanotechnological methodologies. This approach may prove to be an effective means of reducing the potential negative impact on the polymer matrix structure and mechanical properties, especially in the case of biopolymer-based matrices.

Equally important is the assessment of the environmental footprint of EOPE-based packaging through comprehensive life cycle assessments (LCA). Such assessments can help identify opportunities for improvement, such as improving the biodegradability of the films and integrating circular economy principles into their design and disposal. Consumer acceptance studies and market analysis are also critical to understanding the demand for these sustainable packaging solutions and matching their characteristics to consumer expectations.

The ongoing advancements in biotechnology, analytical chemistry, microelectronics, and materials science offer the potential to create innovative, intelligent packaging solutions. Furthermore, the migration of packaging constituents, particularly in the case of nanoparticles, must be subjected to rigorous toxicological analysis and evaluated against the established regulatory limits to ascertain their long-term safety for human health. These developments have the potential to assist in achieving industrial standards for food safety and minimizing the environmental impact, thereby facilitating broader applications of active packaging in the food industry.

## Data Availability

Data not shared.

## Abbreviations Used

ABTS: 2,2′-azinobis-3-ethylbenzothiazoline-6-sulfonic acid
AFM: atomic force microscope
BHA: beta hydroxy acid
BHT: 2,6-di-*tert*-butyl-4-methylphenol
CFU/g: colony-forming unit per gram
DPPH: 2,2-diphenyl-1-picrylhydrazyl
*E. coli*: *Escherichia coli*
EB: elongation at break
EC: European commission
EO: essential oil
EOPE: essential oil Pickering emulsions
FFS: film-forming suspension
GAE: gallic acid equivalent
GRAS: Generally Recognized as Safe
HPMC: hydroxypropyl methylcellulose
LPDE: low-density polyethylene
O/W: oil-in-water emulsion
PE: Pickering emulsion
PVA: poly(vinyl alcohol)
*S. aureus*: *Staphylococcus aureus*
SEM: scanning electron microscope
TBARS: thiobarbituric acid reactive substances
TS: tensile strength
TVB-N: total volatile base nitrogen
TVC: total viable counts
W/O: water-in-oil emulsion
W/O/W: water-in-oil-in-water emulsion
WoS: Web of Science
WPI: whey protein isolate
WVP: vapor transmission

## References

[ref1] FAO (2019) The state food and agriculture. Moving forward on food loss and waste reduction. FAO. Retrieved from http://www.fao.org/3/ca6030en/ca6030en.pdf.

[ref2] Liu Z.; Lin D.; Li N.; Yang X. Characterization of konjac glucomannan-based active films loaded with thyme essential oil: Effects of loading approaches. Food Hydrocolloids 2022, 124 (7), 107330 10.1016/j.foodhyd.2021.107330.

[ref3] Luo L.; Wang M.; Su W.; Zhuo J.; Zhang L.; Zhu W.; Zhang W.; Wang R.; Wang J. Thermal-driven curcumin release film with dual-mode synergistic antibacterial behavior for efficient tangerine preservation. J. Agric. Food Chem. 2024, 72 (3), 1756–1767. 10.1021/acs.jafc.3c07572. 38214269

[ref4] Ran R.; Xiong Y.; Zheng T.; Tang P.; Zhang Y.; Yang C.; Li G. Active and intelligent collagen films containing laccase-catalyzed mulberry extract and Pickering emulsion for fish preservation and freshness indicator. Food Hydrocolloids 2024, 147 (4), 109326 10.1016/j.foodhyd.2023.109326.

[ref5] Roy S.; Rhim J. W. Carrageenan/agar-based functional film integrated with zinc sulfide nanoparticles and Pickering emulsion of tea tree essential oil for active packaging applications. Int. J. Biol. Macromol. 2021, 193, 2038–2046. 10.1016/j.ijbiomac.2021.11.035. 34774596

[ref6] Chamas A.; Moon H.; Zheng J.; Qiu Y.; Tabassum T.; Jang J. H.; Abu-Omar M.; Scott S.; Suh S. Degradation rates of plastics in the environment. ACS Sustainable Chem. Eng. 2020, 8 (9), 3494–3511. 10.1021/acssuschemeng.9b06635.

[ref7] Al-Maqtari Q. A.; Al-Gheethi A. A. S.; Ghaleb A. D.; Mahdi A. A.; Al-Ansi W.; Noman A. E.; Abdulqader A.; Odjo A.; Yuhang D.; Wei M. P.; Yao W. Fabrication and characterization of chitosan/gelatin films loaded with microcapsules of Pulicaria jaubertii extract. Food Hydrocolloids 2022, 129, 107624 10.1016/j.foodhyd.2022.107624.

[ref8] Tian B.; Liu J.; Yang W.; Wan J. B. Biopolymer food packaging films incorporated with essential oils. J. Agric. Food Chem. 2023, 71 (3), 1325–1347. 10.1021/acs.jafc.2c07409. 36628408

[ref9] De Farias P. M.; De Vasconcelos L. B.; Ferreira M. E.; Alves Filho E. G.; Tapia-Blácido D. R. Use of chemically treated nopal cladodes as additive in the cassava starch composite films. J. Vinyl Addit. Technol. 2023, 29 (6), 1109–1124. 10.1002/vnl.22040.

[ref10] Zhang W.; Jiang H.; Rhim J. W.; Cao J.; Jiang W. Effective strategies of sustained release and retention enhancement of essential oils in active food packaging films/coatings. Food Chem. 2022, 367 (1), 130671 10.1016/j.foodchem.2021.130671. 34343816

[ref12] Hosseini S. F.; Zandi M.; Rezaei M.; Farahmandghavi F. Two-step method for encapsulation of oregano essential oil in chitosan nanoparticles: Preparation, characterization and in vitro release study. Carbohydr. Polym. 2013, 95 (1), 50–56. 10.1016/j.carbpol.2013.02.031. 23618238

[ref13] Huang Y.; Liu H.; Liu S.; Li S. Cinnamon cassia oil emulsions stabilized by chitin nanofibrils: Physicochemical properties and antibacterial activities. J. Agric. Food Chem. 2020, 68 (49), 14620–14631. 10.1021/acs.jafc.0c03971. 33226223

[ref14] Tavares L.; Noreña C. P. Z.; Barros H. L.; Smaoui S.; Lima P. S.; de Oliveira M. M. Rheological and structural trends on encapsulation of bioactive compounds of essential oils: A global systematic review of recent research. Food Hydrocolloids 2022, 129 (11), 107628 10.1016/j.foodhyd.2022.107628.

[ref15] Sun H.; Li S.; Chen S.; Wang C.; Liu D.; Li X. Antibacterial and antioxidant activities of sodium starch octenylsuccinate-based Pickering emulsion films incorporated with cinnamon essential oil. Int. J. Biol. Macromol. 2020, 159 (12), 696–703. 10.1016/j.ijbiomac.2020.05.118. 32439447

[ref16] Tavassoli M.; Khezerlou A.; Bangar S. P.; Bakhshizadeh M.; Haghi P. B.; Moghaddam T. N.; Ehsani A. Functionality developments of Pickering emulsion in food packaging: Principles, applications, and future perspectives. Trends Food Sci. Technol. 2023, 132 (3), 171–187. 10.1016/j.tifs.2023.01.007.

[ref17] Almasi H.; Azizi S.; Amjadi S. Development and characterization of pectin films activated by nanoemulsion and Pickering emulsion stabilized marjoram (Origanum majorana L.) essential oil. Food Hydrocolloids 2020, 99, 105338 10.1016/j.foodhyd.2019.105338.

[ref18] Hu J.; Du P.; Xu R.; Deng W. Supersmall dendritic mesoporous silica nanospheres as antioxidant nanocarriers for Pickering emulsifiers. J. Agric. Food Chem. 2021, 69 (49), 14893–14905. 10.1021/acs.jafc.1c03016. 34813315

[ref19] De Farias P. M.; Matheus J. R. V.; Fai A. E. C.; de Vasconcelos L. B.; Tapia-Blácido D. R. Global Research Trends on the Utilization of Nopal (Opuntia Sp) Cladodes as a Functional Ingredient for Industrial Use. Plant Foods Hum. Nutr. 2023, 78 (4), 621–629. 10.1007/s11130-023-01113-2. 37861933

[ref20] Matheus J. R. V.; de Farias P. M.; Satoriva J. M.; de Andrade C. J.; Fai A. E. C. Cassava starch films for food packaging: Trends over the last decade and future research. Int. J. Biol. Macromol. 2023, 225, 658–672. 10.1016/j.ijbiomac.2022.11.129. 36395939

[ref21] Fan S.; Wang D.; Wen X.; Li X.; Fang F.; Richel A.; Xiao N.; Fauconnier M.; Hou C.; Zhang D. Incorporation of cinnamon essential oil-loaded Pickering emulsion for improving antimicrobial properties and control release of chitosan/gelatin films. Food Hydrocolloids 2023, 138 (9), 108438 10.1016/j.foodhyd.2022.108438.

[ref22] Cheng H.; Xu H.; McClements D. J.; Chen L.; Jiao A.; Tian Y.; Miao M.; Jin Z. Recent advances in intelligent food packaging materials: Principles, preparation and applications. Food Chem. 2022, 375, 131738 10.1016/j.foodchem.2021.131738. 34922277

[ref23] Konfo T. R. C.; Djouhou F. M. C.; Koudoro Y. A.; Dahouenon-Ahoussi E.; Avlessi F.; Sohounhloue C. K. D.; Simal-Gandara J. Essential oils as natural antioxidants for the control of food preservation. Food Chemistry Advances 2023, 2 (10), 100312 10.1016/j.focha.2023.100312.

[ref24] Rout S.; Tambe S.; Deshmukh R. K.; Mali S.; Cruz J.; Srivastav P. P.; Amin P.; Gaikwad K.; Andrade E.; de Oliveira M. S. Recent trends in the application of essential oils: The next generation of food preservation and food packaging. Trends Food Sci. Technol. 2022, 129, 421–439. 10.1016/j.tifs.2022.10.012.

[ref25] Ju J.; Chen X.; Xie Y.; Yu H.; Guo Y.; Cheng Y.; Qian H.; Yao W. Application of essential oil as a sustained release preparation in food packaging. Trends Food Sci. Technol. 2019, 92, 22–32. 10.1016/j.tifs.2019.08.005.

[ref26] Fasihi H.; Noshirvani N.; Hashemi M.; Fazilati M.; Salavati H.; Coma V. Antioxidant and antimicrobial properties of carbohydrate-based films enriched with cinnamon essential oil by Pickering emulsion method. Food Packag. Shelf Life 2019, 19, 147–154. 10.1016/j.fpsl.2018.12.007.

[ref27] De Almeida J. M.; Crippa B. L.; de Souza V. V. M. A.; Alonso V. P. P.; Júnior E. D. M. S.; Picone C. S. F.; Prata A.; Silva N. C. C. Antimicrobial action of oregano, thyme, clove, cinnamon and black pepper essential oils free and encapsulated against foodborne pathogens. Food Control 2023, 144 (6), 109356 10.1016/j.foodcont.2022.109356.

[ref28] Eid A. M.; Jaradat N.; Shraim N.; Hawash M.; Issa L.; Shakhsher M.; Nawahda N.; Hanbali A.; Barahmeh N.; Taha B.; Mousa A. Assessment of anticancer, antimicrobial, antidiabetic, anti-obesity and antioxidant activity of Ocimum basilicum seeds essential oil from Palestine. BMC Complementary Med. Ther. 2023, 23 (1), 221 10.1186/s12906-023-04058-w. PMC10321017 37403162

[ref29] Yan J.; Niu Y.; Wu C.; Shi Z.; Zhao P.; Naik N.; Mai X.; Yuan B. Antifungal effect of seven essential oils on bamboo. Adv. Compos. Hybrid Mater. 2021, 4 (3), 552–561. 10.1007/s42114-021-00251-y.

[ref30] Falleh H.; Jemaa M. B.; Saada M.; Ksouri R. Essential oils: A promising eco-friendly food preservative. Food Chem. 2020, 330, 127268 10.1016/j.foodchem.2020.127268. 32540519

[ref31] Ruiz-Navajas Y.; Viuda-Martos M.; Sendra E.; Perez-Alvarez J. A.; Fernández-López J. In vitro antibacterial and antioxidant properties of chitosan edible films incorporated with Thymus moroderi or Thymus piperella essential oils. Food Control 2013, 30 (2), 386–392. 10.1016/j.foodcont.2012.07.052.

[ref32] Delshadi R.; Bahrami A.; Tafti A. G.; Barba F. J.; Williams L. L. Micro and nano-encapsulation of vegetable and essential oils to develop functional food products with improved nutritional profiles. Trends Food Sci. Technol. 2020, 104, 72–83. 10.1016/j.tifs.2020.07.004.

[ref33] Abu Ali O. A.; El-Naggar M. E.; Abdel-Aziz M. S.; Saleh D. I.; Abu-Saied M. A.; El-Sayed W. A. Facile synthesis of natural anise-based nanoemulsions and their antimicrobial activity. Polymers 2021, 13 (12), 2009 10.3390/polym13122009. 34205409 PMC8235015

[ref34] Amor G.; Sabbah M.; Caputo L.; Idbella M.; De Feo V.; Porta R.; Fechtali T.; Mauriello G. Basil essential oil: Composition, antimicrobial properties, and microencapsulation to produce active chitosan films for food packaging. Foods 2021, 10 (1), 121 10.3390/foods10010121. 33430030 PMC7827191

[ref35] Zhang C.; Zhao J.; Famous E.; Pan S.; Peng X.; Tian J. Antioxidant, hepatoprotective and antifungal activities of black pepper (Piper nigrum L.) essential oil. Food Chem. 2021, 346 (1–2), 128845 10.1016/j.foodchem.2020.128845. 33387832

[ref36] El-Baroty G. S.; Abd El-Baky H. H.; Farag R. S.; Saleh M. A. Characterization of antioxidant and antimicrobial compounds of cinnamon and ginger essential oils. Afr. J. Biochem. Res. 2010, 4 (6), 167–174.

[ref37] Verma R. S.; Verma S. K.; Tandon S.; Padalia R. C.; Darokar M. P. Chemical composition and antimicrobial activity of Java citronella (Cymbopogon winterianus Jowitt ex Bor) essential oil extracted by different methods. J. Essent. Oil Res. 2020, 32 (5), 449–455. 10.1080/10412905.2020.1787885.

[ref38] Wu H.; Li J.; Jia Y.; Xiao Z.; Li P.; Xie Y.; Zhang A.; Liu R.; Ren Z.; Zhao M.; Zeng C.; Li C. Essential oil extracted from Cymbopogon citronella leaves by supercritical carbon dioxide: antioxidant and antimicrobial activities. J. Anal. Methods Chem. 2019, 2019 (18), 1–10. 10.1155/2019/8192439. PMC6334621 30719374

[ref39] Bai J.; Li J.; Chen Z.; Bai X.; Yang Z.; Wang Z.; Yang Y. Antibacterial activity and mechanism of clove essential oil against foodborne pathogens. LWT 2023, 173, 114249 10.1016/j.lwt.2022.114249.

[ref40] Alfikri F. N.; Pujiarti R.; Wibisono M. G.; Hardiyanto E. B. Yield, quality, and antioxidant activity of clove (Syzygium aromaticum L.) bud oil at the different phenological stages in young and mature trees. Scientifica 2020, 2020 (4), 1–8. 10.1155/2020/9701701. PMC7290900 32566363

[ref41] Wang X.; Shen Y.; Thakur K.; Han J.; Zhang J. G.; Hu F.; Wei Z. J. Antibacterial activity and mechanism of ginger essential oil against Escherichia coli and Staphylococcus aureus. Molecules 2020, 25 (17), 3955 10.3390/molecules25173955. 32872604 PMC7504760

[ref42] Raspo M. A.; Vignola M. B.; Andreatta A. E.; Juliani H. R. Antioxidant and antimicrobial activities of citrus essential oils from Argentina and the United States. Food Biosci. 2020, 36 (5), 100651 10.1016/j.fbio.2020.100651.

[ref43] Valková V.; Ďúranová H.; Galovičová L.; Vukovic N. L.; Vukic M.; Kačániová M. In Vitro antimicrobial activity of lavender, mint, and rosemary essential oils and the effect of their vapours on growth of Penicillium spp. in a bread model system. Molecules 2021, 26 (13), 3859 10.3390/molecules26133859. 34202776 PMC8270289

[ref44] Valková V.; Ďúranová H.; Galovičová L.; Borotová P.; Vukovic N. L.; Vukic M.; Kačániová M. Cymbopogon citratus essential oil: Its application as an antimicrobial agent in food preservation. Agronomy 2022, 12 (1), 155 10.3390/agronomy12010155.

[ref45] Song X.; Liu T.; Wang L.; Liu L.; Li X.; Wu X. Antibacterial effects and mechanism of mandarin (Citrus reticulata L.) essential oil against Staphylococcus aureus. Molecules 2020, 25 (21), 4956 10.3390/molecules25214956. 33114746 PMC7663016

[ref46] Elshafie S. S.; Elshafie H. S.; El Bayomi R. M.; Camele I.; Morshdy A. E. M. Evaluation of the antimicrobial activity of four plant essential oils against some food and phytopathogens isolated from processed meat products in Egypt. Foods 2022, 11 (8), 1159 10.3390/foods11081159. 35454746 PMC9032107

[ref47] Milenković L.; Ilić Z. S.; Šunić L.; Tmušić N.; Stanojević L.; Stanojević J.; Cvetković D. Modification of light intensity influence essential oils content, composition and antioxidant activity of thyme, marjoram and oregano. Saudi J. Biol. Sci. 2021, 28 (11), 6532–6543. 10.1016/j.sjbs.2021.07.018. 34764769 PMC8568991

[ref48] Lan W.; Zhao X.; Chen M.; Xie J. Antimicrobial activity and mechanism of oregano essential oil against Shewanella putrefaciens. J. Food Saf. 2022, 42 (1), e12952 10.1111/jfs.12952.

[ref49] Luo K.; Zhao P.; He Y.; Kang S.; Shen C.; Wang S.; Guo M.; Wang L.; Shi C. Antibacterial effect of oregano essential oil against Vibrio vulnificus and its mechanism. Foods 2022, 11 (3), 403 10.3390/foods11030403. 35159553 PMC8834123

[ref50] Mutlu-Ingok A.; Catalkaya G.; Capanoglu E.; Karbancioglu-Guler F. Antioxidant and antimicrobial activities of fennel, ginger, oregano and thyme essential oils. Food Front. 2021, 2 (4), 508–518. 10.1002/fft2.77.

[ref51] Saraiva C.; Silva A. C.; García-Díez J.; Cenci-Goga B.; Grispoldi L.; Silva A. F.; Almeida J. M. Antimicrobial activity of Myrtus communis L. and Rosmarinus officinalis L. essential oils against Listeria monocytogenes in cheese. Foods 2021, 10 (5), 1106 10.3390/foods10051106. 34067614 PMC8156628

[ref52] Yang Z.; Li M.; Li Y.; Li Z.; Huang X.; Wang X.; Shi J.; Xiaobo Z.; Xiaodong Z.; Povey M.; Xiao J. Improving properties of Litsea cubeba oil Pickering emulsion-loaded gelatin-based bio-nanocomposite film via optimizing blending ratio: Application for mango preservation. Food Hydrocolloids 2023, 145 (2), 109052 10.1016/j.foodhyd.2023.109052.

[ref53] Low L. E.; Siva S. P.; Ho Y. K.; Chan E. S.; Tey B. T. Recent advances of characterization techniques for the formation, physical properties and stability of Pickering emulsion. Adv. Colloid Interface Sci. 2020, 277, 102117 10.1016/j.cis.2020.102117. 32035999

[ref54] Badmus S. O.; Amusa H. K.; Oyehan T. A.; Saleh T. A. Environmental risks and toxicity of surfactants: overview of analysis, assessment, and remediation techniques. Environ. Sci. Pollut. Res. 2021, 28 (44), 62085 10.1007/s11356-021-16483-w. PMC8480275 34590224

[ref55] Campolo O.; Giunti G.; Laigle M.; Michel T.; Palmeri V. Essential oil-based nano-emulsions: Effect of different surfactants, sonication and plant species on physicochemical characteristics. Ind. Crops Prod. 2020, 157, 112935 10.1016/j.indcrop.2020.112935.

[ref56] Ming L.; Wu H.; Liu A.; Naeem A.; Dong Z.; Fan Q.; Zhang G.; Liu H.; Li Z. Evolution and critical roles of particle properties in Pickering emulsion: A review. J. Mol. Liq. 2023, 388 (14), 122775 10.1016/j.molliq.2023.122775.

[ref57] De Farias P. M.; Matheus J. R. V.; Maniglia B. C.; Le-Bail P.; Le-Bail A.; Schmid M.; Fai A. E. C. Bibliometric mapping analysis of Pickering emulsion applied in 3D food printing. Int. J. Food Sci. Technol. 2024, 59 (4), 2186–2196. 10.1111/ijfs.17040.

[ref58] Jiang Q.; Binks B. P.; Meng Z. Double scaffold networks regulate edible Pickering emulsion gel for designing thermally actuated 4D printing. Food Hydrocolloids 2022, 133 (7), 107969 10.1016/j.foodhyd.2022.107969.

[ref59] Griffith C.; Daigle H. A comparison of the static and dynamic stability of Pickering emulsions. Colloids Surf., A 2020, 586, 124256 10.1016/j.colsurfa.2019.124256.

[ref60] Li C.; Xie W.; Zhang X.; Liu J.; Zhang M.; Shao J. H. Pickering emulsion stabilized by modified pea protein-chitosan composite particles as a new fat substitute improves the quality of pork sausages. Meat Sci. 2023, 197 (1–2), 109086 10.1016/j.meatsci.2022.109086. 36580792

[ref61] Yi J.; Gan C.; Wen Z.; Fan Y.; Wu X. Development of pea protein and high methoxyl pectin colloidal particles stabilized high internal phase Pickering emulsions for β-carotene protection and delivery. Food Hydrocolloids 2021, 113 (5), 106497 10.1016/j.foodhyd.2020.106497.

[ref62] Ahsan H. M.; Pei Y.; Luo X.; Wang Y.; Li Y.; Li B.; Liu S. Novel stable Pickering emulsion based solid foams efficiently stabilized by microcrystalline cellulose/chitosan complex particles. Food Hydrocolloids 2020, 108 (2), 106044 10.1016/j.foodhyd.2020.106044.

[ref63] Dai L.; Zhan X.; Wei Y.; Sun C.; Mao L.; McClements D. J.; Gao Y. Composite zein-propylene glycol alginate particles prepared using solvent evaporation: Characterization and application as Pickering emulsion stabilizers. Food Hydrocolloids 2018, 85, 281–290. 10.1016/j.foodhyd.2018.07.013.

[ref64] Nimaming N.; Sadeghpour A.; Murray B. S.; Sarkar A. Hybrid particles for stabilization of food-grade Pickering emulsions: Fabrication principles and interfacial properties. Trends Food Sci. Technol. 2023, 138 (1), 671–684. 10.1016/j.tifs.2023.06.034.

[ref65] Wu H.; Wang J.; Li T.; Lei Y.; Peng L.; Chang J.; Li S.; Yuan X.; Zhou M.; Zhang Z. Effects of cinnamon essential oil-loaded Pickering emulsion on the structure, properties and application of chayote tuber starch-based composite films. Int. J. Biol. Macromol. 2023, 240 (10), 124444 10.1016/j.ijbiomac.2023.124444. 37062380

[ref66] Deng N.; Hu Z.; Li H.; Li C.; Xiao Z.; Zhang B.; Liu M.; Fang F.; Wang J.; Cai Y. Physicochemical properties and pork preservation effects of lotus seed drill core powder starch-based active packaging films. Int. J. Biol. Macromol. 2024, 260, 129340 10.1016/j.ijbiomac.2024.129340. 38262831

[ref67] Yao L.; Man T.; Xiong X.; Wang Y.; Duan X.; Xiong X. HPMC films functionalized by zein/carboxymethyl tamarind gum stabilized Pickering emulsions: Influence of carboxymethylation degree. Int. J. Biol. Macromol. 2023, 238 (1), 124053 10.1016/j.ijbiomac.2023.124053. 36934825

[ref68] Zhao Z.; Liu H.; Tang J.; He B.; Yu H.; Xu X.; Li C.; Wang C.; Liu Y.; Su Y.; Chen S. Pork preservation by antimicrobial films based on potato starch (PS) and polyvinyl alcohol (PVA) and incorporated with clove essential oil (CLO) Pickering emulsion. Food Control 2023, 154 (23), 109988 10.1016/j.foodcont.2023.109988.

[ref69] Punia Bangar S.; Whiteside W. S.; Ozogul F.; Dunno K. D.; Cavender G. A.; Dawson P. Development of starch-based films reinforced with cellulosic nanocrystals and essential oil to extend the shelf life of red grapes. Food Biosci. 2022, 47 (4), 101621 10.1016/j.fbio.2022.101621.

[ref70] Zhang S.; He Z.; Xu F.; Cheng Y.; Waterhouse G. I.; Sun-Waterhouse D.; Wu P. Enhancing the performance of konjac glucomannan films through incorporating zein–pectin nanoparticle-stabilized oregano essential oil Pickering emulsions. Food Hydrocolloids 2022, 124, 107222 10.1016/j.foodhyd.2021.107222.

[ref71] Liang W.; Ge X.; Lin Q.; Niu L.; Zhao W.; Muratkhan M.; Li W. Ternary composite degradable plastics based on Alpinia galanga essential oil Pickering emulsion templates: A potential multifunctional active packaging. Int. J. Biol. Macromol. 2024, 257 (5), 128580 10.1016/j.ijbiomac.2023.128580. 38052283

[ref72] Liu J.; Song F.; Chen R.; Deng G.; Chao Y.; Yang Z.; Wu H.; Bai M.; Zhang P.; Hu Y. Effect of cellulose nanocrystal-stabilized cinnamon essential oil Pickering emulsions on structure and properties of chitosan composite films. Carbohydr. Polym. 2022, 275 (1), 118704 10.1016/j.carbpol.2021.118704. 34742429

[ref73] Zhao R.; Guan W.; Zheng P.; Tian F.; Zhang Z.; Sun Z.; Cai L. Development of edible composite film based on chitosan nanoparticles and their application in packaging of fresh red sea bream fillets. Food Control 2022, 132 (1), 108545 10.1016/j.foodcont.2021.108545.

[ref74] Zhao R.; Guan W.; Zhou X.; Lao M.; Cai L. The physiochemical and preservation properties of anthocyanidin/chitosan nanocomposite-based edible films containing cinnamon-perilla essential oil Pickering nanoemulsions. LWT 2022, 153 (2), 112506 10.1016/j.lwt.2021.112506.

[ref75] Roy S.; Priyadarshi R.; Rhim J. W. Gelatin/agar-based multifunctional film integrated with copper-doped zinc oxide nanoparticles and clove essential oil Pickering emulsion for enhancing the shelf life of pork meat. Food Res. Int. 2022, 160 (2), 111690 10.1016/j.foodres.2022.111690. 36076394

[ref76] Wang J.; Sun X.; Zhang H.; Dong M.; Li L.; Zhangsun H.; Wang L. Dual-functional intelligent gelatin based packaging film for maintaining and monitoring the shrimp freshness. Food Hydrocolloids 2022, 124 (17), 107258 10.1016/j.foodhyd.2021.107258.

[ref77] Yudhistira B.; Chang C. K.; Punthi F.; Cheng K. C.; Husnayain N.; Hsieh C. W. Application of LDPE film–loaded nutmeg essential oil Pickering emulsion to extend tilapia fillets’ shelf life. Food Bioprocess Technol. 2024, 17 (10), 3031–3045. 10.1007/s11947-023-03306-8.

[ref78] Guo X.; Wang X.; Wei Y.; Liu P.; Deng X.; Lei Y.; Zhang J. Preparation and properties of films loaded with cellulose nanocrystals stabilized Thymus vulgaris essential oil Pickering emulsion based on modified tapioca starch/polyvinyl alcohol. Food Chem. 2024, 435 (3), 137597 10.1016/j.foodchem.2023.137597. 37797451

[ref79] Zomorodian N.; Javanshir S.; Shariatifar N.; Rostamnia S. The effect of essential oil of Zataria multiflora incorporated chitosan (free form and Pickering emulsion) on microbial, chemical and sensory characteristics in salmon (Salmo trutta). Food Chem.: X 2023, 20 (1), 100999 10.1016/j.fochx.2023.100999. 38144780 PMC10740042

[ref80] Zhao P.; Yan X.; Cheng M.; Wang Y.; Wang Y.; Wang K.; Xiangyou W.; Wang J. Effect of Pickering emulsion on the physical properties, microstructure and bioactivity of corn starch/cassia gum composite films. Food Hydrocolloids 2023, 141 (1), 108713 10.1016/j.foodhyd.2023.108713.

[ref81] Wigati L. P.; Wardana A. A.; Tanaka F.; Tanaka F. Application of pregelatinized corn starch and basil essential oil edible coating with cellulose nanofiber as Pickering emulsion agent to prevent quality-quantity loss of mandarin orange. Food Packag. Shelf Life 2023, 35 (1), 101010 10.1016/j.fpsl.2022.101010.

[ref82] Fasihi H.; Noshirvani N.; Hashemi M. Novel bioactive films integrated with Pickering emulsion of ginger essential oil for food packaging application. Food Biosci. 2023, 51 (2), 102269 10.1016/j.fbio.2022.102269.

[ref83] Nkede F. N.; Wardana A. A.; Phuong N. T. H.; Takahashi M.; Koga A.; Wardak M. H.; Fanze M.; Tanaka F.; Tanaka F. Preparation and characterization of chitosan/lemongrass oil/cellulose nanofiber Pickering emulsions active packaging and its application on tomato preservation. J. Polym. Environ. 2023, 31 (11), 4930–4945. 10.1007/s10924-023-02885-z.

[ref84] Ju J.; Xie Y.; Yu H.; Guo Y.; Cheng Y.; Zhang R.; Yao W. Synergistic inhibition effect of citral and eugenol against Aspergillus niger and their application in bread preservation. Food Chem. 2020, 310 (4), 125974 10.1016/j.foodchem.2019.125974. 31835216

[ref85] Chaudhari A. K.; Singh V. K.; Das S.; Dubey N. K. Nanoencapsulation of essential oils and their bioactive constituents: A novel strategy to control mycotoxin contamination in food system. Food Chem. Toxicol. 2021, 149, 112019 10.1016/j.fct.2021.112019. 33508419

[ref86] Hosseini S. F.; Ghaderi J.; Gómez-Guillén M. C. Tailoring physico-mechanical and antimicrobial/antioxidant properties of biopolymeric films by cinnamaldehyde-loaded chitosan nanoparticles and their application in packaging of fresh rainbow trout fillets. Food Hydrocolloids 2022, 124 (5), 107249 10.1016/j.foodhyd.2021.107249.

[ref89] Khwaldia K.; Attour N.; Matthes J.; Beck L.; Schmid M. Olive byproducts and their bioactive compounds as a valuable source for food packaging applications. Compr. Rev. Food Sci. Food Saf. 2022, 21 (2), 1218–1253. 10.1111/1541-4337.12882. 35068049

